# Interplay between Ca^2+^/Calmodulin-Mediated Signaling and AtSR1/CAMTA3 during Increased Temperature Resulting in Compromised Immune Response in Plants

**DOI:** 10.3390/ijms23042175

**Published:** 2022-02-16

**Authors:** Peiguo Yuan, B. W. Poovaiah

**Affiliations:** Department of Horticulture, Washington State University, Pullman, WA 99164-6414, USA; pomology2010@gmail.com

**Keywords:** AtSR1/CAMTA3, calcium signaling, stomatal immunity, SA signaling, temperature

## Abstract

Changing temperatures are known to affect plant–microbe interactions; however, the molecular mechanism involved in plant disease resistance is not well understood. Here, we report the effects of a moderate change in temperature on plant immune response through Ca^2+^/calmodulin-mediated signaling. At 30 °C, *Pst* DC3000 triggered significantly weak and relatively slow Ca^2+^ influx in plant cells, as compared to that at 18 °C. Increased temperature contributed to an enhanced disease susceptibility in plants; the enhanced disease susceptibility is the result of the compromised stomatal closure induced by pathogens at high temperature. A Ca^2+^ receptor, AtSR1, contributes to the decreased plant immunity at high temperatures and the calmodulin-binding domain (CaMBD) is required for its function. Furthermore, both salicylic acid biosynthesis (ICS) and salicylic acid receptor (NPR1) are involved in this process. In addition to stomatal control, AtSR1 is involved in high temperature-compromised apoplastic immune response through the salicylic acid signaling pathway. The qRT-PCR data revealed that AtSR1 contributed to increased temperatures-mediated susceptible immune response by regulating SA-related genes in *atsr1*, such as *PR1*, *ICS1*, *NPR1*, as well as *EDS1*. Our results indicate that Ca^2+^ signaling has broad effects on the molecular interplay between changing temperatures as well as plant defense during plant–pathogen interactions.

## 1. Introduction

Extreme temperatures cause adverse impacts on plant growth and development, which can lead to significant crop losses all over the world [1]. In recent years, global warming has resulted in more frequent extreme temperature events [2]. Plant disease is another major cause of agricultural loss [3,4,5]. Environmental temperature changes antagonistically interact with the plant immune response, for example, plant immunity is usually repressed at high temperatures, as compared to low temperatures [3,6,7,8]. However, the molecular mechanisms involved in plant responses at varying temperatures have remained elusive. 

Plants use hormones to adapt to various environmental stimuli, including pathogen infections [9]. Salicylic acid (SA) acts as one of the main defense phytohormones against biotrophic and hemi-biotrophic microbes in both local and systemic resistance [10]. Facing the pathogen challenges, plants establish immune resistance through reprograming SA-related genes, (such as Enhanced Disease Susceptibility 1 (EDS1), Phytoalexin Deficient 4 (PAD4), Isochorismate Synthase 1 (ICS1) and *Nonexpresser of Pathogenesis-Related Genes 1 (NPR1)*) and activating the biosynthesis of SA [10,11]. SA also plays a role in the association between plant immune response and temperature. For instance, the accumulation of SA induced by pathogen was compromised at 30 °C, and the gene expressions of *PAD4* and *EDS1* were reduced at higher temperatures [12]. Unlike SA signals, temperature regulated jasmonate (JA) and/or ethylene (ET) act in an opposing manner [13]. JA and/or ET signaling was suppressed at low temperature, while high temperature promoted JA and/or ET-mediated plant immunity [14,15,16]. 

In addition to phytohormone-regulated defense, high temperature also reduced resistance (R) protein (which are nucleotide binding-leucine rich repeat, NB-LRR, protein) mediated immune response in plants [17,18]. Pathogens secrete effectors into plant cells to suppress the innate immune response and improve their virulence, however, the microbe-derived effector is recognized by R protein [19]. The recognition and activation of R protein results in a rapid and strong resistance (also known as effector-triggered immunity, ETI), and sometimes associated with programmed cell death at the infected site, termed hypersensitive response (HR) [19]. At high temperature, ETI or HR was inhibited [20]. *Pseudomonas syringae* pv. Tomato (*Pst*) DC3000 carrying AvrRpt2, AvrRmp1 and AvrRps4 induced HR in *Arabidopsis* at 22 °C, however, the ETI-HR was inhibited at 28 °C [6]. Several mutants are also reported to display temperature-sensitive autoimmune phenotypes, such as enhanced SA accumulation, retarded growth, and constitutively activated defense pathways. Some mutants, such as *Bonzai 1* (*bon1*) [12], *Suppressor of Npr1 Constitutive 1* (*snc1*) [12], and *Mapk/Erk Kinase Kinase 1* (*mekk1*) [21] display the autoimmune phenotype at normal temperature (22 °C), which was compromised at a higher temperature (28 °C); whereas, other mutants, such as *Suppressor of Salicylic acid Insensitive 4* (*ssi4*) [22], *chilling-sensitive 2 (chs2)* [23], *Ler/Kashmir 2* (*kas-2*) [24], and *Uk1* [25] display the autoimmune phenotype only at low temperatures (14–16 °C), which was compromised at normal temperature (22 °C). Additionally, the increased temperature repressed the temperature-dependent autoimmunity phenotype in some mutated plants, such as *atsr1* or *rps4-OE* [26,27]. 

Guard cells represent one of the most significant cell types in terrestrial plants, which forms the microscopic pores in the epidermis to ensure gas (CO_2_ and O_2_) exchange and transpiration [28]. Plants regulate the movement of guard cells by ABA in response to drought and/or salt stress [29]. Some studies have revealed that the stomatal opening and closure are essential for plant immune response, since the stomates were found to act as the invasion entry into the leaf interior for a large number of bacterial pathogens [30,31,32]. Plants detect the pathogen-associated molecular patterns (PAMPs) to induce stomatal closure to prevent pathogen entry [30,32]. The PAMPs preceptors (such as FLS2) and the pathogen-triggered accumulation of SA are required for the pathogen-induced stomatal closure [32]. Meanwhile, virulent pathogens generate coronatine (COR), which is structurally similar to jasmonoyl-isoleucine (JA-Ile), to suppress SA-regulated stomatal immunity through activated JA signaling [32]. Bacterial pathogens have evolved with type III secretion systems to deliver the effector into the plant cell to suppress PTI, which is also known as effector-triggered susceptibility (ETS) [19]. The expression of HopM1 (an effector protein from *Pst* DC3000) in *Arabidopsis*-compromised flg22 triggered production of reactive oxygen species (ROS) and stomatal closure [33]. In addition to stomatal closure, a novel mechanism of stomatal immunity was revealed where the stomal cell death was triggered to prevent the pathogen invasion; a recent study revealed that *Arabidopsis* plants can sense fungal chitin to induce guard cell death to evade fungal infection [30]. Chitin also induced guard cell movement through Ca^2+^ and Calcium-dependent Protein Kinases (CPKs), such as CPK6 [30].

Calcium ion (Ca^2+^) acts as a second messenger in plant cells and is responsible for a large number of environmental stimuli and developmental cues [34,35,36]. Calcium signaling plays a vital role in sensing environmental stimuli and establishing a proper response to maintain optimal growth and development [37,38,39]. The role of Ca^2+^ signaling in plant defense has been well documented. Plants employ Pattern Recognition Receptors (PRRs) to sense the Microbe-Associated Molecular Patterns (MAMP)s and trigger Ca^2+^ influx into plant cells [40,41,42]. Effectors from pathogens can induce an increase in Ca^2+^ influx as compared to MAMPs [11]. CPKs/CDPKs activate TFs to induce transcriptional expression of defense-related genes [43,44]. Furthermore, CaM-binding transcription factors are known to regulate SA signaling [45,46] as well as temperature-modulated Ca^2+^ signaling [47]. Low temperature is known to induce Ca^2+^ influx in roots [48]. In recent years, it has become clear that a change in temperature induces a rise in cytosolic Ca^2+^. Cold stress is known to induce a rise in Ca^2+^ in plant cells, whereas heat stress increases the free Ca^2+^ concentration in the chloroplast, but not in the cytoplast [49,50]. In addition, a chloroplast-specific Ca^2+^ rise was also found in the light to dark transition [51].

Although Ca^2+^ signaling mediates temperature stress and immune response, the role of Ca^2+^ signaling in temperature-mediated plant immune response needs further research to better understand the underlying mechanisms involved in this process. Here, we report that the inoculation with Pst DC3000 induced a strong Ca^2+^ spike in plant cells, while the pathogen-triggered Ca^2+^ spike was compromised to some extent at high temperatures. High temperatures repressed plant immunity, and AtSR1 is involved in this process. Furthermore, through the SA signaling pathway AtSR1 also plays a role in increased plant disease susceptibility at high temperatures.

## 2. Results

### 2.1. Ca*^2+^* Influx by Pathogen Was Repressed at High Temperature

**Figure 1:**
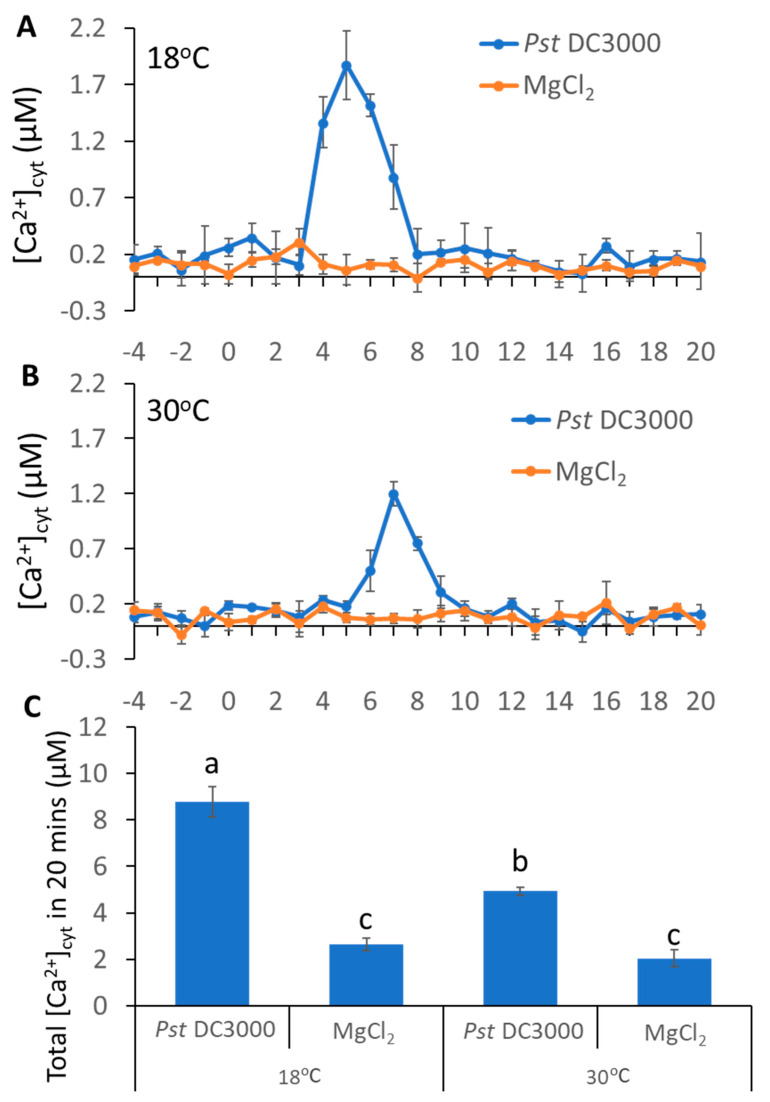
**Pathogen-induced different Ca^2+^ spikes in plants grown at different temperatures.** (**A**,**B**), pathogen-triggered cytosolic Ca^2+^ elevation in leaves of *aequorin*-expressing *Arabidopsis* plants. The dip inoculation of *Pst* DC3000 (OD = 0.01) for leaf discs at 18 °C (**A**) and 30 °C (**B**). Results shown are mean values ± SD (*n* = 4). (**C**) The histogram shows total [Ca^2+^]_cyt_ 20 min after pathogen addition. The data were analyzed by a two-factor ANOVA with all pairs Tukey’s HSD post hoc analysis (*p* < 0.05) for statistical tests: different letters indicate statistical significance; samples sharing letters are not significantly different at 18 or 30 °C.

Our previous study revealed that temperature can affect plant immune response [26]. To investigate whether the inoculation with pathogens triggered different Ca^2+^ influxes at different temperatures, we used WT plants carrying *aequorin (AEQ)* grown at 18 or 30 °C. At the lower temperature, pathogens induced an increase in Ca^2+^ concentration at 4 min post inoculation and reached the peak at 5 min; whereas at 30 °C, pathogens induced a rise of Ca^2+^ at 6 min post inoculation and reached the peak at 7 min (Figure 1A,B). Further analysis revealed that pathogens triggered nearly a two-fold increase in Ca^2+^ influx at low temperature, as compared to that at high temperature (Figure 1C). These observations suggest that the increased temperature delayed and decreased the pathogen-induced Ca^2+^ spikes.

### 2.2. AtSR1 Contributed to the Suppression of High Temperature-Mediated Changes in Stomatal Aperture

**Figure 2:**
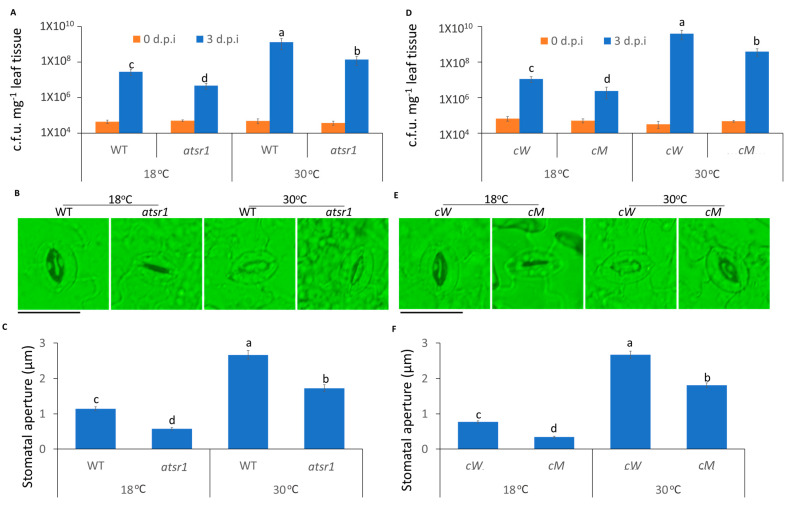
**The stomatal immunity was compromised at high temperature and AtSR1 contributed to high temperature-mediated increased plant disease susceptibility**. (**A**) *Arabidopsis* WT and *atsr1* mutant plants were dip inoculated with *Pst* DC3000 (OD600 = 0.1). The colony forming units (c.f.u.) were calculated at 0 and 3 d.p.i. The growth of *Pst* DC3000 in WT and *atsr1* at 18 °C and 30 °C, respectively, are shown. Error bars represent standard error of six biological repeats. (**B**) Photograph shows the stomatal movement induced by the dip inoculation of *Pst* DC3000 (OD600 = 0.1) in WT and *atsr1* at different temperatures. The scale bar represents 20 μM. (**C**) Stomatal apertures were determined in *Arabidopsis* WT and *atsr1* mutant plants 1 h after incubation of *Pst* DC3000 (OD600 = 0.1). (**D**) *Arabidopsis cW* and *cM* mutant plants were dip inoculated with *Pst* DC3000 (OD600 = 0.1). The colony forming units (c.f.u.) were calculated at 0 and 3 d.p.i. The growth of *Pst* DC3000 in *cW* and *cM* at 18 and 30°C, respectively, is shown. Error bars represent standard error of six biological repeats. (**E**) Photo shows the stomatal movement induced by the incubation of *Pst* DC3000 (OD600 = 0.1) in *cW* and *cM* at different temperatures. The scale bar represents 20 μM. (**F**) Stomatal aperture in *Arabidopsis cW* and *cM* mutant plants 1 h after incubation of *Pst* DC3000 (OD600 = 0.1). All data are representative as means s.e.m. from four independent experiments. The data were analyzed by a two-factor ANOVA with all pairs Tukey’s HSD post hoc analysis (*p* < 0.05) for statistical tests: different letters indicate statistical significance; samples sharing letters are not significantly different at 18 or 30 °C.

Ca^2+^ changes induced by pathogens lead to stomatal closure, which prevents pathogen entry into plants [52]. Thus, we used dip inoculation to assess the effect of temperature on stomatal immunity. At 3 days post inoculation (3 d.p.i), the propagation of pathogens was greatly increased at high temperature, as compared to low temperature (Figure 2A). To further examine whether temperature regulated stomatal changes by pathogen infection, we inoculated peeled plant leaf sections with the pathogen. To rule out the possibility that the different temperatures lead to the different stomatal apertures, we exposed the peeled leaf samples at strong light for 3 h at 18 and 30 °C to ensure that the stomates were completely opened before inoculation (Figures S1 and S2). Consistent with disease resistance data, the width of the stomatal aperture was decreased at low temperature 1 h after inoculation, as compared to high temperature (Figure 2B,C). 

In our previous studies, AtSR1 was reported to act as a Ca^2+^/CaM-mediated transcription factor to regulate plant defense signaling [26]. Therefore, we hypothesized that temperature-regulated stomatal movement involves AtSR1. *Atsr1* mutant displayed more resistance to pathogen attack at 18 °C, as compared to WT (Figure 2A); while the enhanced resistance in *atsr1* was partially maintained at 30 °C (Figure 2A), suggesting that AtSR1 modulates high temperature-promoted plant disease susceptibility. Furthermore, the average width of stomatal aperture was significantly decreased in *atsr1*, as compared to WT, at both 18 and 30 °C (Figure 2B,C). Our results suggest that AtSR1 contributes to high temperature-mediated compromised stomatal immunity.

It was shown that the calmodulin-binding domain (CaMBD) of AtSR1 is required for the suppression of AtSR1 during plant immunity [11]. To further test whether CaMBD is required for AtSR1-mediated stomatal immunity at different temperatures, two complemented lines were used: *cW* (expressing WT AtSR1 in *atsr1* mutant) and *cM* (expressing mutant AtSR1 in *atsr1* mutant K907E at CaMBD, which lacks the CaM-binding ability). As shown in Figure 1D–F, the complemented line, *cW*, was restored to WT *Arabidopsis* with regards to high temperature-induced disease susceptibility and wider stomata aperture. In contrast, *cM* plants displayed the phenotype of the loss-of-function of AtSR1 (Figure 1D–F). These results indicate that the disruption of CaMBD in AtSR1 compromises its mediation in temperature-regulated stomatal defense.

### 2.3. AtSR1 Contributes to Decreased Stomatal Immunity at 30 °C in a SA-Dependent Manner

**Figure 3:**
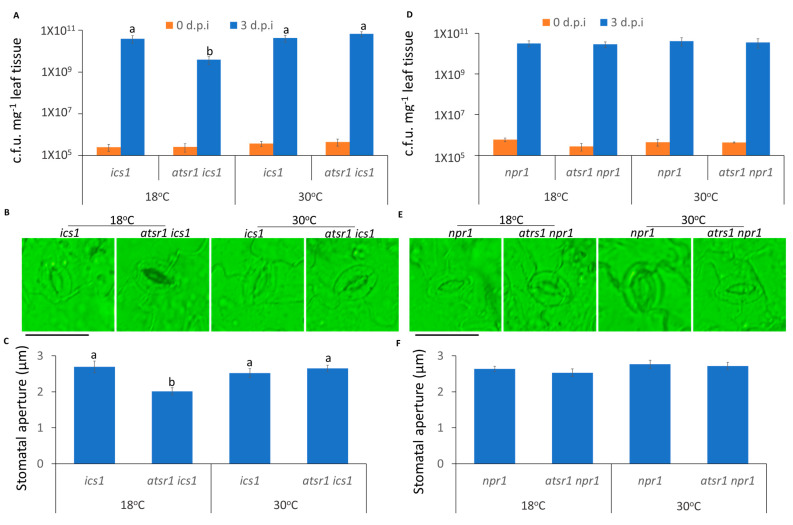
**AtSR1 contributed to high temperature-mediated repressed stomatal immunity in a SA-dependent manner.** (**A**) *Arabidopsis ics1* and *atsr1 ics1* mutant plants were dip inoculated with *Pst* DC3000 (OD600 = 0.1). The colony forming units (c.f.u.) were calculated at 0 and 3 d.p.i. The growth of *Pst* DC3000 in *ics1* and *atsr1 ics1* at 18 and 30 °C, respectively, is shown. Error bars represent standard error of six biological repeats. (**B**) Photograph shows the stomatal movement induced by the incubation of *Pst* DC3000 (OD600 = 0.1) in *ics1* and *atsr1 ics1* at different temperatures. The scale bar represents 20 μM. (**C**) Stomatal aperture in *Arabidopsis ics1* and *atsr1 ics1* mutant plants 1 h after incubation of *Pst* DC3000 (OD600 = 0.1). (**D**) The *Arabidopsis npr1* and *atsr1 npr1* mutant plants were dip inoculated with *Pst* DC3000 (OD600 = 0.1). The colony forming units (c.f.u.) were calculated at 0 and 3 d.p.i. The growth of *Pst* DC3000 in *npr1* and *atsr1 npr1* at 18 and 30 °C, respectively, is shown. Error bars represent standard error of six biological repeats. (**E**) Photograph shows the stomatal movement induced by the incubation of *Pst* DC3000 (OD600 = 0.1) in *npr1* and *atsr1 npr1* at different temperatures. The scale bar represents 20 μM. (**F**) Stomatal aperture in *Arabidopsis npr1* and *atsr1 npr1* mutant plants 1 h after incubation of *Pst* DC3000 (OD600 = 0.1). All data is representative as means s.e.m. from four independent experiments. The data were analyzed by a two-factor ANOVA with all pairs Tukey’s HSD post hoc analysis (*p* < 0.05) for statistical tests: different letters indicate statistical significance; samples sharing letters are not significantly different at 18 or 30 °C.

We next tested the requirement of SA signaling and its role in the AtSR1-mediated stomatal immunity at different temperatures. ICS1 was reported to be a key enzyme for SA biosynthesis in *Arabidopsis*. The growth of pathogen was repressed in *atsr1 ics1* double mutant at low temperature, as compared to *ics1* mutant (Figure 3A); whereas, the decreased pathogen growth in *atsr1 ics1* double mutant was compromised at high temperature (Figure 3A). Similarly, the average width of the stomatal aperture was decreased in the double mutant at low temperature (Figure 3B,C). However, at 30 °C, the average width of the stomatal aperture was not significantly decreased in the double mutant as compared to *ics1* single mutant (Figure 3B,C). This suggests that the biosynthesis of SA is required for AtSR1-mediated reduced stomatal immunity at high temperature.

The SA receptor, NPR1, is also an important component of SA-regulated plant immunity [53,54]. Unlike *ics1*, the enhanced resistance in *atsr1* was compromised in the *npr1* mutant background (Figure 3A–C), indicating that the SA receptor is necessary for AtSR1-involved compromised stomatal immunity at 30 °C, as compared to 18 °C.

### 2.4. The Suppression of Plant Apoplastic Immunity at Increased Temperature Is AtSR1-Dependent

**Figure 4:**
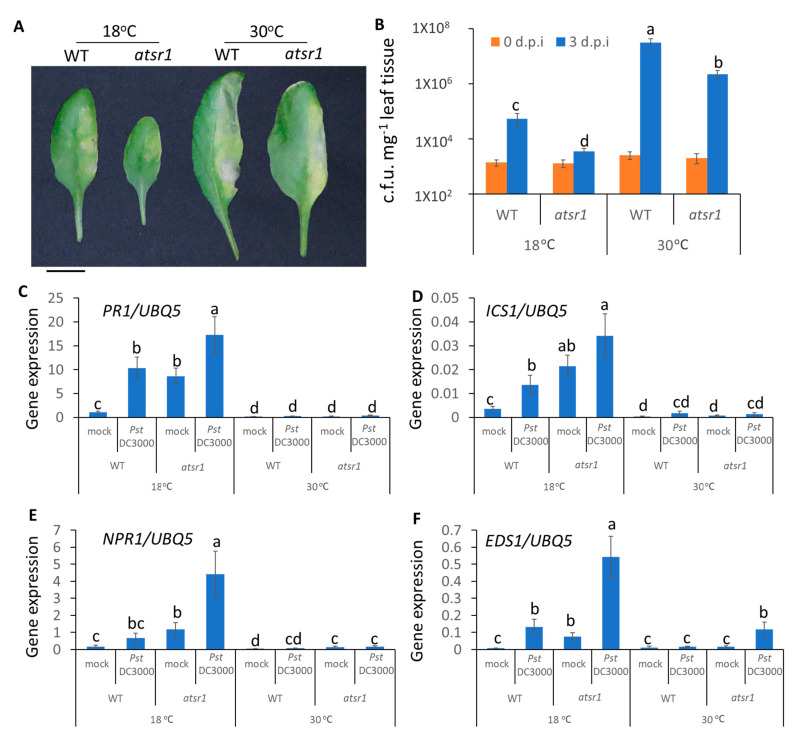
**AtSR1 mediates increased temperature-promoted susceptibility in plant apoplast immunity.** (**A**) Photograph shows the disease symptoms of rosette leaves in WT and *atsr1* mutant plants at 3 days post inoculation (d.p.i.) with the infiltrating inoculation of *Pst* DC3000 (OD600 = 0.001). The scale bar represents 1cM. (**B**) The colony forming units (c.f.u.) were calculated at 0 and 3 d.p.i. The growth of *Pst* DC3000 in WT and *atsr1* at 18 and 30 °C, respectively, is shown. Error bars represent standard error of six biological repeats. (**C**–**F**) Pathogen induced the expression of defense genes in WT and *atsr1* at both 18 and 30 °C. *PR1* (**C**), *ICS1* (**D**), *NPR1* (**E**), and ESD1 (**F**) at 1 day post inoculation of *Pst* DC3000 (OD600 = 0.001) *AtUBQ5* was used as an internal control. All data were representative as means s.e.m. from four independent experiments. The data were analyzed by a two-factor ANOVA with Tukey’s HSD post hoc analysis (*p* < 0.05) for statistical tests: different letters indicate statistical significance; samples sharing letters are not significantly different at 18 or 30 °C.

In addition to the control of pathogen entry, we further determined the temperature-mediated plant immunity to restrict the propagation of pathogens. To test the role of AtSR1 in plant apoplastic immune response, we used the infiltrating inoculation of the pathogen to rule out the effect of AtSR1 on stomatal closure. As shown in Figure 4A, *atsr1* was more resistant to pathogen infection at low temperature, as compared to WT; there is decreased bacterial growth in *atsr1*, as compared to WT (Figure 4B). Interestingly, the enhanced disease resistant phenotype in *atsr1* was partially retained at 30 °C (Figure 4A,B), which indicates that AtSR1 contributes to high temperature-promoted disease susceptibility. 

Previous studies have revealed that AtSR1 suppresses the transcriptional expression of SA-related genes which are involved in plant immune response at 20 °C [11,40]. Hence, we determined the transcriptional expressions of SA-related genes at different temperatures. At 18 °C, pathogens significantly induced *PR1* expression in *atsr1*, as compared to WT, and the constitutively induced PR1 expression in *atsr1* is pathogen-independent (Figure 4C). The enhanced *PR1* expression in *atsr1* was compromised at 30 °C. Similar results were observed in the *ICS1* and *NPR1* expressions (Figure 4D,E). Earlier studies have revealed that the nucleotide-binding domain leucine-rich repeat (NLR)-signaling was also involved in temperature-mediated plant immunity [8]. Hence, we measured the transcriptional expression of *EDS1*, regulated by NLR signaling, which was reported to be regulated by AtSR1 [26]. The expression of *EDS1* induced by pathogens was regulated by AtSR1 at both 18 and 30 °C (Figure 4F). 

### 2.5. CaMBD Is Required for the Regulation of AtSR1 for Enhanced Apoplastic Disease Susceptibility at 30 °C

**Figure 5:**
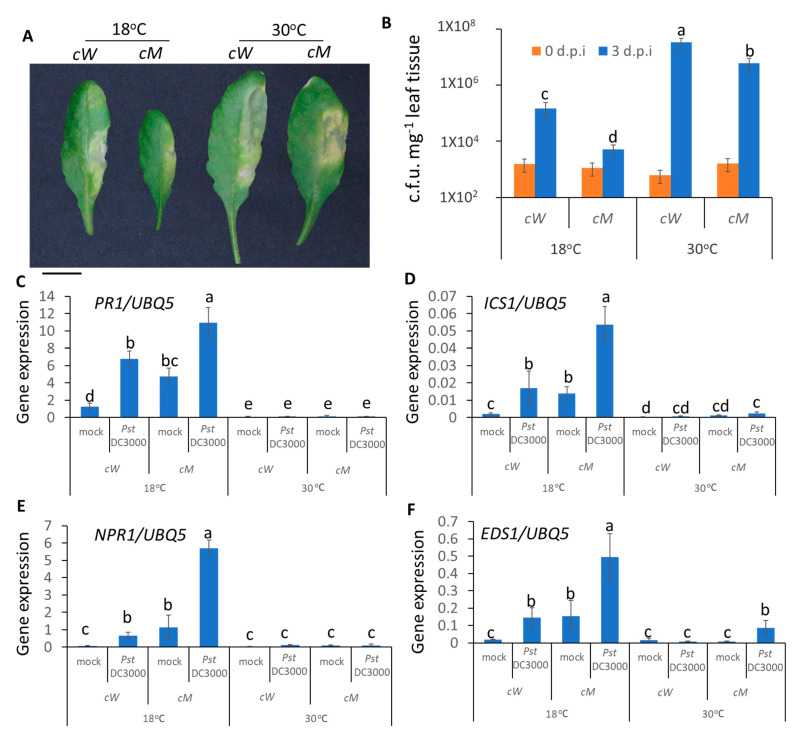
**CaMBD in AtSR1 is required for AtSR1-mediated decreased plant apoplast immune response at high temperature.** (**A**) Photographs show the disease symptoms of rosette leaves in *cW* and *cM* mutant plant at 3 days post inoculation (d.p.i.) with the infiltrating inoculation of *Pst* DC3000 (OD600 = 0.001). The scale bar represents 1cM. (**B**) The colony forming units (c.f.u.) were calculated at 0 and 3 d.p.i. The growth of *Pst* DC3000 in *cW* and *cM* at 18 and 30 °C, respectively, is shown. Error bars represent standard error of six biological repeats. (**C**–**F**) Pathogen induced the expression of defense genes in WT and *atsr1* at both 18 and 30 °C. *PR1* (**C**), *ICS1* (**D**), *NPR1* (**E**), and *ESD1* (**F**) at 1 day post inoculation of *Pst* DC3000 (OD600 = 0.001). *AtUBQ5* was used as an internal control. All data were representative as means s.e.m. from four independent experiments. The data were analyzed by a two-factor ANOVA with Tukey’s HSD post hoc analysis (*p* < 0.05) for statistical tests: different letters indicate statistical significance; samples sharing letters are not significantly different at 18 or 30 °C.

We further tested the role of CaMBD in the AtSR1-regulated temperature-dependent apoplastic immune response. As shown in Figure 5, the complemented line, *cW*, was restored to WT *Arabidopsis* with regards to high temperature-induced susceptibility and the transcriptional expression of defense-related genes, such as *PR1*, *ICS1*, *NPR1*, and *EDS1*. In contrast, *cM* plants displayed the phenotype of the loss-of-function of AtSR1 (Figure 5). These observations suggest that the CaMBD in AtSR1 is essential for its involvement in high temperature-mediated compromised apoplastic immune response in plants.

### 2.6. Involvement of AtSR1 in Enhanced Apoplastic Susceptibility at Increased Temperature Is Dependent on SA Signaling

**Figure 6:**
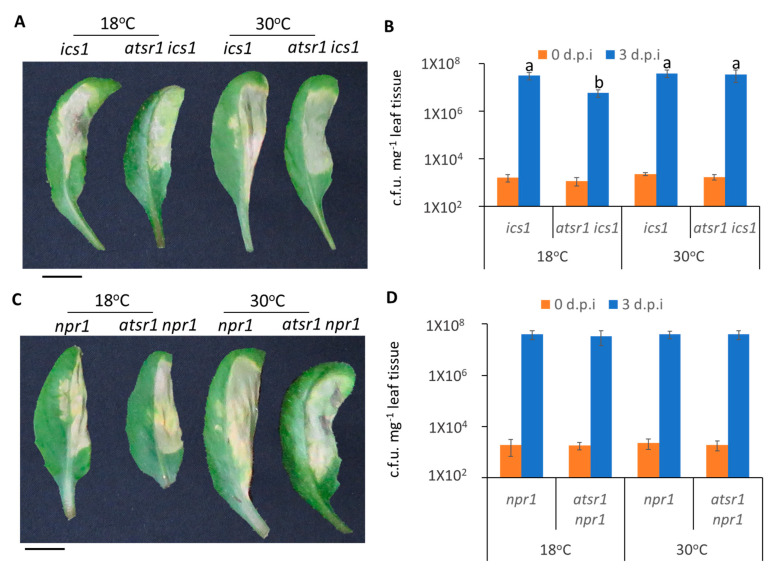
**AtSR1 is involved in high temperature-regulated compromised apoplastic immunity in plants through the SA-signaling pathway**. (**A**) Photograph shows the disease symptoms of rosette leaves in *ics1* and *astsr1 ics1* mutant plant at 3 days post inoculation (d.p.i.) with the infiltrating inoculation of *Pst* DC3000 (OD600 = 0.001). The scale bar represents 1cM. (**B**) The colony forming units (c.f.u.) were calculated at 0 and 3 d.p.i. The growth of *Pst* DC3000 in *ics1* and *astsr1 ics1* at 18 and 30 °C, respectively, is shown. Error bars represent standard error of six biological repeats. (**C**) The photograph shows the disease symptoms of rosette leaves in *npr1* and *astsr1 npr1* mutant plant at 3 days post inoculation (d.p.i.) with the infiltrating inoculation of *Pst* DC3000 (OD600 = 0.001). The scale bar represents 1cM. (**D**) The colony forming units (c.f.u.) were calculated at 0 and 3 d.p.i. The growth of *Pst* DC3000 in *npr1* and *astsr1 npr1* at 18 and 30 °C, respectively, is shown. Error bars represent standard error of six biological repeats. The data were analyzed by a two-factor ANOVA with Tukey’s HSD post hoc analysis (*p* < 0.05) for statistical tests: different letters indicate statistical significance; samples sharing letters or no letter are not significantly different at 18 or 30 °C.

To test whether SA signaling is required for AtSR1-regulated plant apoplastic defense at different temperatures, we carried out disease resistance assays using infiltration inoculation in *atsr1 ics1* and *atsr1 npr1* double mutants. We observed that *atsr1 ics1* displayed improved resistance as compared to *ics1* single mutants at 18 °C, however, the improved resistant phenotype was compromised at high temperature (Figure 6A,B). Unlike the mutants of *ics1*, *atsr1 npr1* double mutant plants displayed similar resistance as compared to *npr1* single mutant plants at both 18 and 30 °C (Figure 6C,D), indicating that AtSR1 regulates increased apoplastic susceptibility at high temperature in a SA-dependent manner. 

## 3. Discussion

Based on the 50-year-old concept of the “disease triangle”, successful survival of plants facing pathogen attack requires established effective immune response, suppressed pathogen virulence, and favorable environmental conditions [55]. Hence, our goal was to further investigate the plant–microbe interactions at varying temperatures to extend our knowledge of plant immune response.

Temperature is one of the most important environmental factors which impacts plant growth and development [47,56]. In addition, ambient temperature is a major contributor to plant immunity and growth regulation [57]. Temperature also influences plant and microbe interactions [58]. Plants sense pathogen attack, which triggers Ca^2+^ influx in the plant cell [59,60]. Plants regulate a complicated network of signaling pathways to establish immune responses to pathogen invasion; Ca^2+^ signaling cascade is a key determinant for plants to integrate the various environmental stimuli to prevent invading pathogens [42,61]. However, whether pathogens at different temperatures induce different Ca^2+^ influxes are not clearly understood. In this study, we observed that the rise of Ca^2+^ in plant cells triggered by pathogen is greatly reduced at high temperature, and the time to reach the highest Ca^2+^ flux was slightly delayed as compared to low temperature (Figure 1). This indicates a possible connection between the increased susceptibility at high temperature and compromised Ca^2+^ signaling, although further studies are needed in this area. Previous studies have supported the observation that both of the increased basal and increased flg22-induced Ca^2+^ concentration in the *aca4/11* double mutant, as compared to WT, were suppressed at high temperature [62]. In addition, AtACA4 and AtACA11 were identified as two tonoplast-localized Ca^2+^ pumps. The reasonable explanation is that the altered temperature affects the Ca^2+^ pumps or Ca^2+^ channel to regulate Ca^2+^ influx during plant–microbe interaction. Another possible explanation is that the compromised rise of Ca^2+^ at high temperature is possibly due to the increase in the free Ca^2+^ concentration within the stroma of chloroplasts [50]. However, a reduced Ca^2+^ spike in the chloroplast caused by a decrease in SA accumulation subsequently reduced SA-related gene expressions [63], although the underlying mechanisms of these interactions are still not clear.

It is becoming clear that stomates are the natural entry point for bacterial pathogens into plants and that Ca^2+^ signaling plays a critical role in regulating the stomatal immunity. A recent study reported that reduced hyperosmolality-induced [Ca^2+^]i increase 1.3 (OSCA1.3), acted as a Ca^2+^-permeable channel which regulates stomatal closure during pathogen infection [64]. In addition, two-pore channel 1 (TPC1), known as Ca^2+^-dependent Ca^2+^-release channel localized in the vacuole, regulates stomatal movement [65]. The stomatal closure was impaired in *tpc1*, suggesting Ca^2+^ influx is necessary for stomatal closure to stop the entry of pathogens into plants [65]. Our results indicate that the increased temperature repressed the stomatal immunity. The average width of stomatal apertures was increased at 30 °C, as compared to 18 °C (Figure 2). Moreover, the growth of pathogens in plants was greatly increased at 30 °C when we used dip inoculation to mimic plants facing pathogen attack in nature. Previous studies have revealed that AtSR1 is a suppressor of plant defense signaling and is involved in abiotic stress, especially cold stress [66,67]. Our results further confirm that AtSR1 is also a suppressor of stomatal immunity. The reduced growth of pathogens was tested in *atsr1*; moreover, reduced width of stomatal aperture was observed in the mutants (Figure 2). Hence, these results suggest that AtSR1 contributes to high temperature-mediated enhanced stomatal susceptibility. 

Typically, the intact and functional CaMBD plays a critical role in CaM-binding proteins [68,69]. The mutated CaMBD in CaM-binding proteins leads to a loss-of-function protein. Previous studies have revealed that in mutated AtSR1 (K907E), a single amino acid mutated at the CaMBD failed to bind to calmodulin and the *atsr1* mutants complemented with mutated AtSR1 (K907E, *cM*) resembled the *atsr1* plants, but not WT *Arabidopsis*. Similarly, the mutated complemented line, *cM*, displayed increased resistance and reduced width of stomatal aperture (Figure 3D–F). In addition, the temperature-regulated stomatal response was partially retained in *cM* mutated plants. This observation indicates that Ca^2+^/CaM-binding is required for the function of AtSR1 to suppress stomatal immunity at high temperatures.

The defense phytohormone, SA, plays a key role in stomatal immunity [70]. The stomatal closure induced by pathogens was impaired in WT carrying NahG, encoding salicylate hydroxylase that converts SA to catechol [32]. In this study, we observed that SA signaling is required for AtSR1-regulated stomatal immunity (Figure 3). Unlike *atsr1*, *atsr1 ics1* double mutant displayed a decreased plant immune response in stomates, and increased width of stomatal aperture triggered by pathogen (Figure 3). Our results are consistent with previous studies that indicate that high temperature promoted pathogen susceptibility in plants through the suppression of SA accumulation [71,72]. In addition to SA biosynthesis, we also tested the SA receptor, NPR1. We observed a similar disease symptom in *atsr1 npr1* double mutant. These results suggest that the production of SA is required for AtSR1-mediated stomatal immunity, and the SA receptor, NPR1, is also required. 

In plant innate immune response, stomatal immunity contributed to limiting the pathogen invasion into the plant and apoplastic immunity repressed the growth and propagation of the pathogens after the entry into the plants [73]. In addition to temperature-mediated stomatal immunity, we determined that the temperature-mediated apoplastic immune response is required for AtSR1. As is the case for apoplastic immunity, *atsr1* mutant is more resistant to pathogen attack at 30 °C, as compared to WT; the pathogen propagation was also repressed in *atsr1* at 30 °C (Figure 4). These results suggest that AtSR1 regulates enhanced temperature-promoted plant susceptibility. Moreover, increased temperatures decreased the induction of SA-biosynthesis genes, such as *EDS1*, *PAD4*, and *ICS1*, resulting in reduced plant defense against pathogen as compared to that in low temperature [71,72]. Our results support the previous study that the enhanced expression of SA-related genes (*PR1*, *ICS1*, and *NPR1*) at 18 °C were compromised at 30 °C in *atsr1*, indicating that AtSR1 regulates enhanced susceptibility at high temperature in a SA-dependent way (Figure 4). Ca^2+^ and calmodulin play a key role in AtSR1-regulated temperature-depended plant immune response, as shown by similar results observed in *cM* as compared to *cW* (Figure 5). However, apoplastic immunity is not always consistent with stomatal immunity. A previous study identified some mutants, such as *scord2* and *scord4*, which displayed normal stomatal defense, but reduced apoplastic immune response [74]. 

Previous studies have revealed that the accumulation of SA is required for high temperature to confer the suppression of plant immune response. Our observation further confirms that AtSR1 protein represses plant immune response at 30 °C in a SA-dependent manner, resulting in increased plant immunity at high temperature in *atsr1*, which was compromised in *ics1* background mutants (Figure 6). Our studies also indicate that SA biosynthesis as well as the SA receptor, NPR1, are required for AtSR1-mediated temperature-dependent plant immune response (Figure 6). Both *atsr1* and the *suppressor of npr1*, *constitutive 1* (*snc1*) mutant plants displayed a temperature-sensitive autoimmunity phenotype. The temperature sensitive autoimmunity phenotype in *snc1* was compromised in *Phytochrome Interacting Factor 4* (*pif4*) or *Sap and Miz1 domain-containing Ligase 1* (*siz1*) mutants, respectively [75,76], which raises a question about whether PIF4- and/or SIZ1 mediated the autoimmunity phenotype in *atsr1*. 

## 4. Methods and Materials

### 4.1. Plant Materials and Growth Conditions

The genetic resources for this study are wild-type (WT) Columbia (Col-0), loss-of-function *atsr1* mutant (Salk_001152C), loss-of-function *ics1* mutant (Salk_088254), and loss-of-function *npr1* line (Salk_204100C), which were ordered from ABRC; as well as complimentary AtSR1 lines in *atsr1*, i.e., *cW* and *cM* (K907E) which were generated in a previous study [26]. The homozygous knock-out mutants were verified by PCR and RT-PCR. 

The seeds were surface sterilized with 10% diluted bleach for 5 min and then 70% ethanol for another 5 min. The sterilized seeds were washed 5 times with sterilized water and placed on half-strength MS medium (Caisson Laboratories Inc., Smithfield, UT, USA) containing 0.05% MES and 1% sucrose, adjusting pH to 5.7 with KOH at 4 °C dark for 3 days and germinated in a growth chamber (humidity 60–70%) under 12 h light (light condition: 100–150 μE·m^−2^·s^−1^) and dark periods at 18 or 30 °C, respectively. One-week-old seedlings were transferred to pots containing soil mix (Metro Mix 360 Rsi, Sun Gro Horticulture, Agawam, MA, USA). Plants were maintained in a growth chamber under a 12 h photoperiod at 18 °C for low temperature or 30 °C for high temperature (humidity: 60%; light condition: 100–150 μE·m^−2^·s^−1^).

### 4.2. Calcium Measurement

The calcium spikes in leaves were measured with AEQ-based calcium assay [77,78,79]. The *Arabidopsis* Col-0 plants carrying *AEQ* were grown in soil at 18 or 30 °C. The leaf discs (5 mm diameter) obtained from 4-week-old plants were immersed into 1 mL of 5 μM coelenterazine solution (NanoLight Technologies, Aurora, CO, USA) in 24-well microplates. The plate was left under vacuum for 10 min twice, and then further incubated overnight in the dark at 18 or 30 °C, respectively. The AEQ-based bioluminescence was quantified in a microplate reader for 5 min as baseline. An equal volume of double-strength pathogen was added (the final concentration of *Pst* DC3000 is OD600 = 0.01) and quantified for 20 min, as L (luminescence intensity per second). The total remaining Ca^2+^ in each microplate well was discharged by treatment with equal volume of 2 M CaCl_2_ in 20% ethanol to release remaining AEQ, as L_max_. Ca^2+^ concentrations in plant cells were calculated as described previously (Tanaka et al., 2013). The equation is: [Ca^2+^]_cyt_ (nM) = [X + (X∗55) − 1]/(1 − X)/0.02, where X = (L/L_max_)1/3.

### 4.3. Disease Resistance Assay

*Pst* DC3000 was cultured in King’s B medium (20 g/L peptone, 1.5 g/L MgSO_4_, 1.5 g/L K_2_HPO_4_, pH = 7), containing 50 ug/mL rifamycin and 25 ug/mL kanamycin, overnight at 28 °C. The cells were harvested (until an OD600 = 0.5 was reached) by centrifugation (10,000× *g*, for 10 min), washed twice by autoclaved water, and diluted into the desired density as described below.

For dip inoculation: the cells were diluted to OD600 of 0.1, containing 0.05% Silwet L-77 in 10 mM MgCl_2_. The 4- or 5-week-old WT and mutated *Arabidopsis* plants were dipped in bacterial suspension with gentle shaking for 5 min and kept under high humidity in the dark overnight. At 1 h after inoculation (as day 0) and 3 days after inoculation (as 3 d.p.i, at 18 or 30 °C, respectively), the leaf samples were harvested for disease resistance test. The leaf samples were weighed and ground in 1 mL autoclaved water; serial dilutions were dropped on King’s B medium containing antibiotic as described above. Then, 48 h after being grown at 28 °C, the bacterial colony forming units (c.f.u.) were calculated.

For infiltrated inoculation: the leaf inoculation was performed as previously described [34]. Briefly, the pathogens were prepared as described above. Leaves of 4- to 5-week-old plants were infiltrated with *Pst* DC3000 at OD600 of 0.001 in 10 mM MgCl_2_, using 1 mL needleless syringe for time course induction (for testing the gene expression of defense-related genes) and disease resistance test. At 1 h after inoculation (as day 0) and 3 days after inoculation (as 3 d.p.i., at 18 or 30 °C, respectively), the leaf samples were harvested for disease resistance test. The leaf sample was weighed and ground in 1 mL autoclaved water; serial dilutions were dropped on King’s B medium containing antibiotic as descripted above. Then, 48 h after growth at 28 °C, the bacterial colony forming units (c.f.u.) were calculated. Data were shown as an average of six biological replicates; the results are presented as mean ± S.D.

### 4.4. Measurements of Stomatal Aperture

Stomatal aperture was measured in epidermal peels excised from the abaxial side of leaves of 4- or 5-week-old WT or mutated *Arabidopsis* plants described previously [80]. In order to ensure that all genotypes of the plants at different temperature had almost fully open stomata, the peeled leaf samples were incubated at 10mL stomatal opening solution (30 mM KCl, 1 mM CaCl_2_, 10 mM Tris, pH 5.8) to expose to white light (150–200 μE·m^−2^·s^−1^) for at least 3 h, at 18 or 30 °C, respectively. An equal volume (10 mL) of pathogen (OD600 = 0.2, in 10 mM MgCl_2_) was added into the peeled leaf samples with stomatal opening solution, at 18 or 30 °C, respectively. The stomatal movement was imaged and counted using light microscopy (200×). The stomatal aperture was measured using ImageJ software. Data are calculated as the average of 10 stomatal aperture for the leaf sample from 4 individual seedlings.

### 4.5. RNA Extraction and Transcriptional Expression Analysis

Four-week-old WT and mutated *Arabidopsis* seedlings were used to measure gene expressions. At one day post inoculation, 100 mg leaf tissues were harvested from control and infected leaf samples at different temperatures of different genotypes and immediately frozen in liquid nitrogen. The frozen tissues were ground to powder in 1.5 mL Eppendorf tubes. Total RNA was prepared using TRIzol Reagent (Invitrogen) based on the manufacturer’s protocol, followed by DNase-I (Roche) treatment. In total, 2 µg total RNA were used to synthesize cDNA with an oligo (dT) primer and random hexadeoxynucleotides primer (Thermo Fisher Scientific, Waltham, MA, USA). The cDNA was diluted 10 times and 1 μL/reaction (10 μL) was used as a template. Real-time PCR was performed on a MyiQTM single-color real-time PCR detection system using SYBR Green Supermix (Bio-Rad). Target gene expression levels were normalized to that of *AtUBQ5* (AT3G62250). A minimum of two technical replicates and four biological replicates were used for each sample.

### 4.6. Data Analysis

Results were analyzed using Microsoft Excel. Error bars in all of the figures represent standard error [81]. Number of replicates is described in the figure legends. For multiple group samples, statistical analyses were performed by two-way ANOVA with Tukey’s HSD (honest significant difference) test. The different letters (a, b, c) indicate samples with statistically significant differences (*p* < 0.05), while the same letter indicates no statistically significant difference.

## 5. Conclusions

Our results suggest that Ca^2+^ transients triggered by pathogen were compromised at high temperature. High temperature contributed to enhanced plant susceptibility in both stomatal defense and apoplastic immune response. AtSR1/CAMTA3, as a Ca^2+^/CaM receptor, is involved in increased temperature-mediated stomatal defense and apoplastic immunity. In addition, the contribution of AtSR1 to the temperature-modulated plant immune response requires functional CaMBD in AtSR1. This and other studies indicate that Ca^2+^ signaling acts as a general defense response to pathogen infection in the context of temperature.

## Supplementary Materials

Supplementary materials can be found at https://www.mdpi.com/article/10.3390/ijms23042175/s1.
