# Supplementary material for: Interplay between Ca2+/Calmodulin-Mediated Signaling and AtSR1/CAMTA3 during Increased Temperature Resulting in Compromised Immune Response in Plants
Source: Int J Mol Sci. 2022 Feb 16;23(4):2175. doi: 10.3390/ijms23042175 (PMC8880272; doi:10.3390/ijms23042175)
Supplement: Supplementary file 1 [file ijms-23-02175-s001.zip › ijms-1594951-supplementary.pdf]

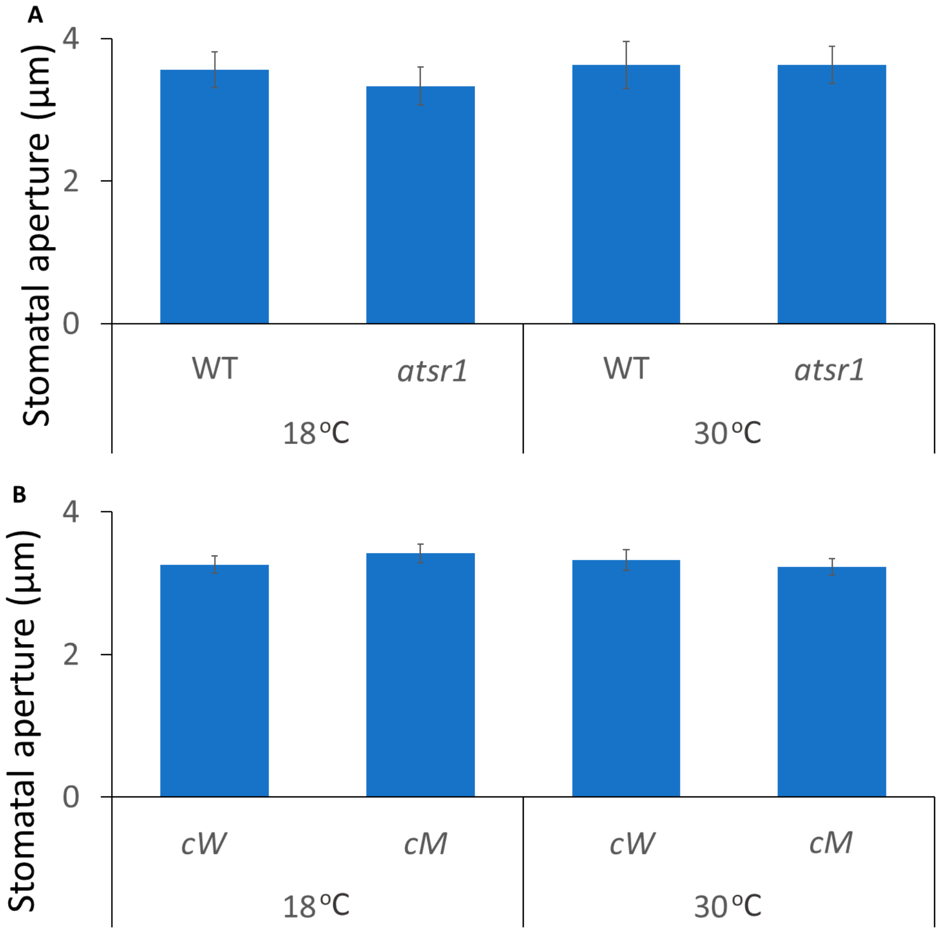

Figure S1. (A) The figure shows the stomatal aperture in non-inoculated leaf tissue in WT and *atsr1* at 18 or 30 °C. (B) The figure shows the stomatal aperture in non-inoculated leaf tissue from *cW* and *cM* at 18 or 30 °C, respectively.

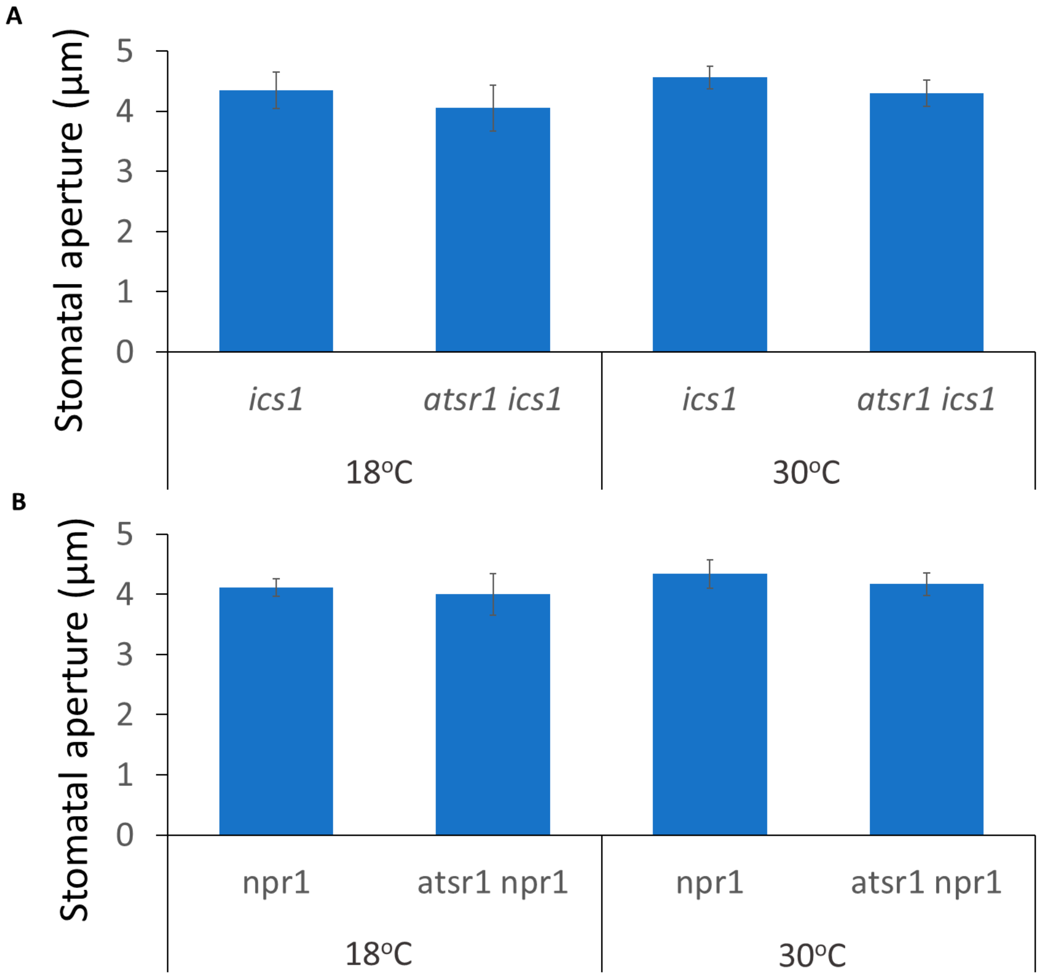

Figure S2. (A) The figure shows the stomatal aperture in non-inoculated leaf tissue from *ics1* and *atsr1 ics1* at different temperatures. (B) The figure shows the stomatal aperture in non-inoculated leaf tissue from *npr1* and *atsr1 npr1* at different temperatures.
